# Supplementary material for: Pathomechanism Characterization and Potential Therapeutics Identification for Parkinson’s Disease Targeting Neuroinflammation
Source: Int J Mol Sci. 2021 Jan 21;22(3):1062. doi: 10.3390/ijms22031062 (PMC7865530; doi:10.3390/ijms22031062)
Supplement: Supplementary file 1 [file ijms-22-01062-s001.zip › original blot images 1,21.pptx]

## Slide 1
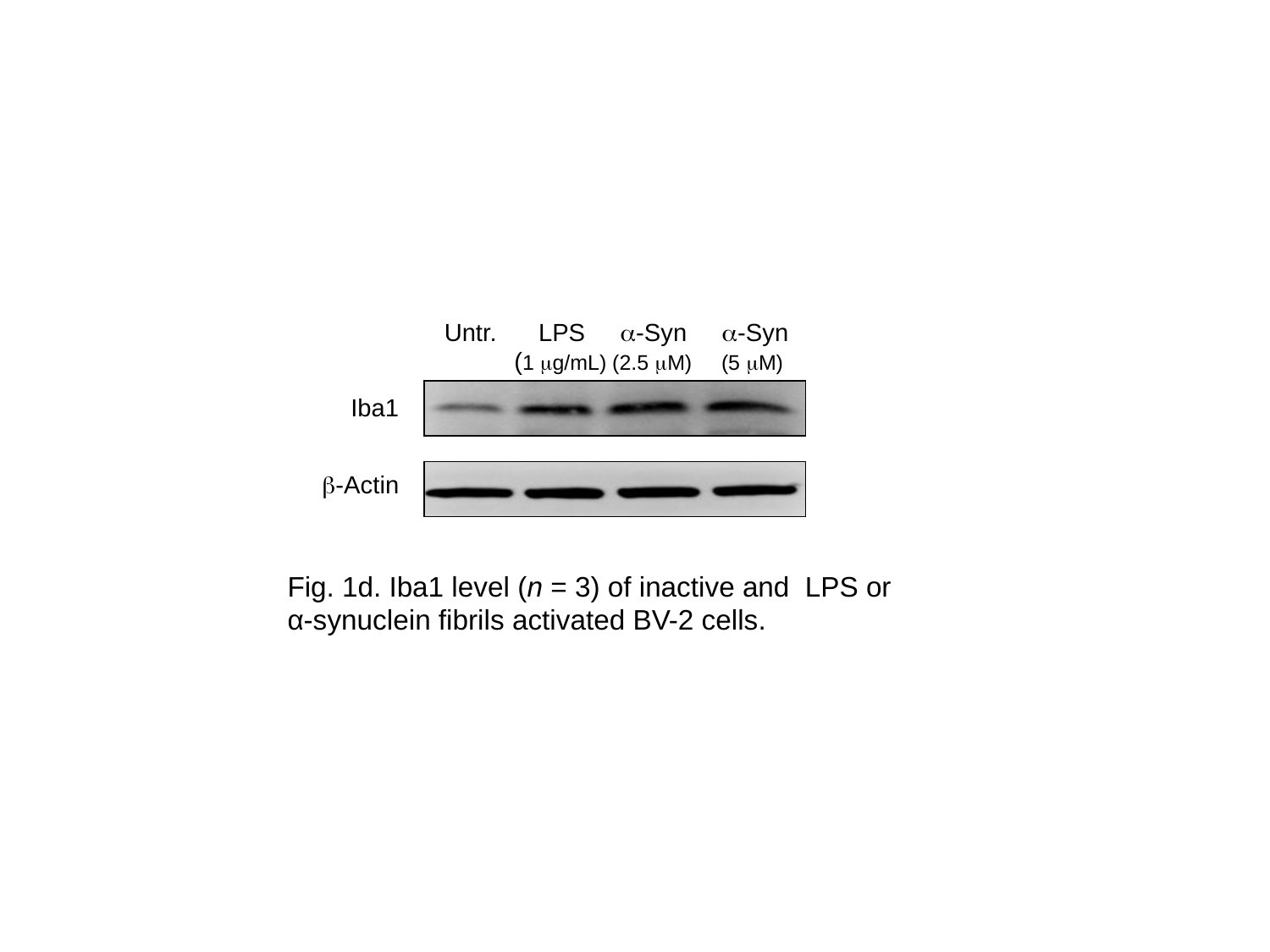

Untr. LPS a-Syn a-Syn
 (1 mg/mL) (2.5 mM) (5 mM)
Iba1
b-Actin
Fig. 1d. Iba1 level (n = 3) of inactive and LPS or α-synuclein fibrils activated BV-2 cells.

## Slide 2
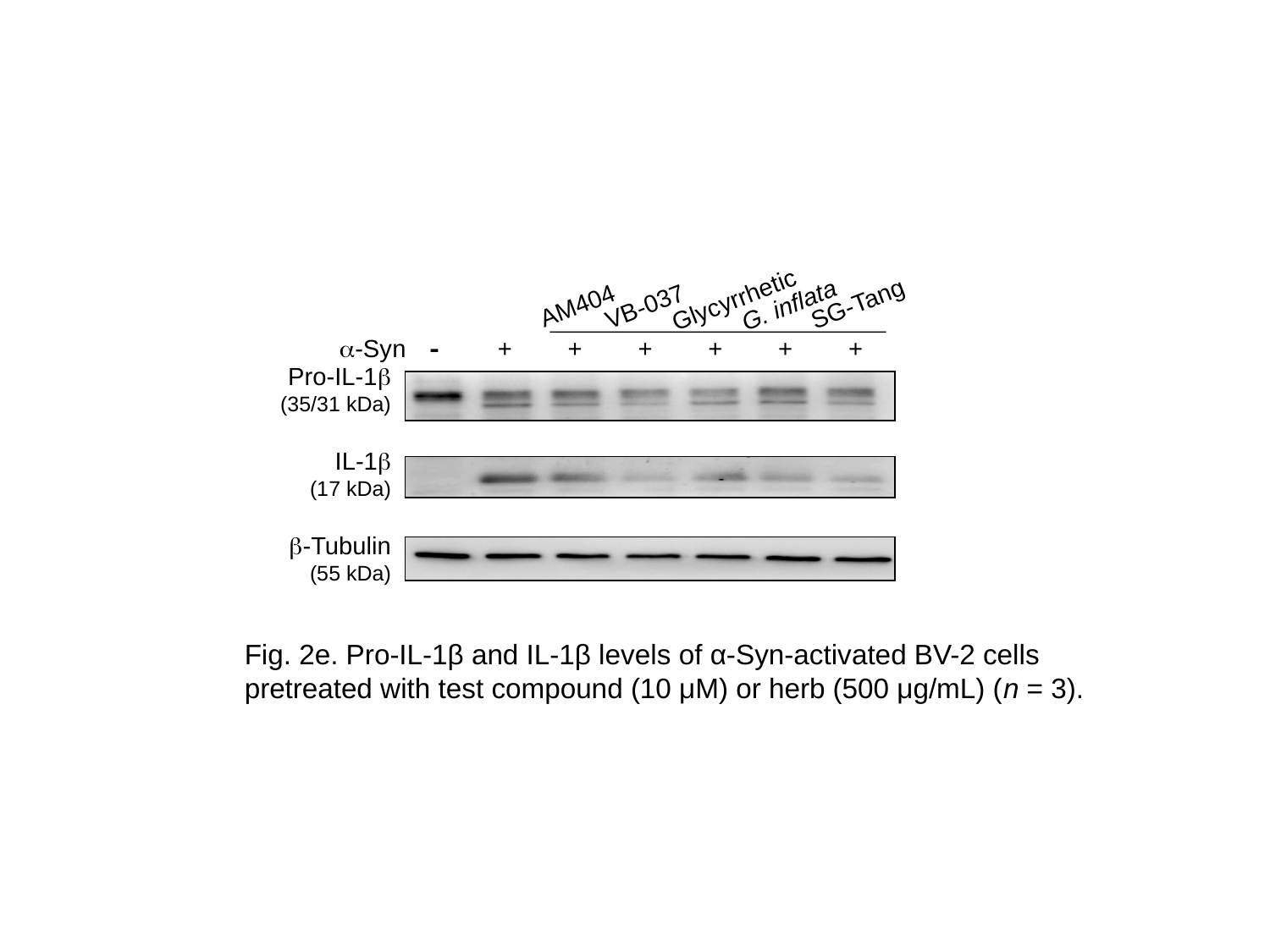

G. inflata
Glycyrrhetic
SG-Tang
AM404
VB-037
a-Syn - + + + + + +
Pro-IL-1b
(35/31 kDa)
IL-1b
(17 kDa)
b-Tubulin
(55 kDa)
Fig. 2e. Pro-IL-1β and IL-1β levels of α-Syn-activated BV-2 cells pretreated with test compound (10 μM) or herb (500 μg/mL) (n = 3).

## Slide 3
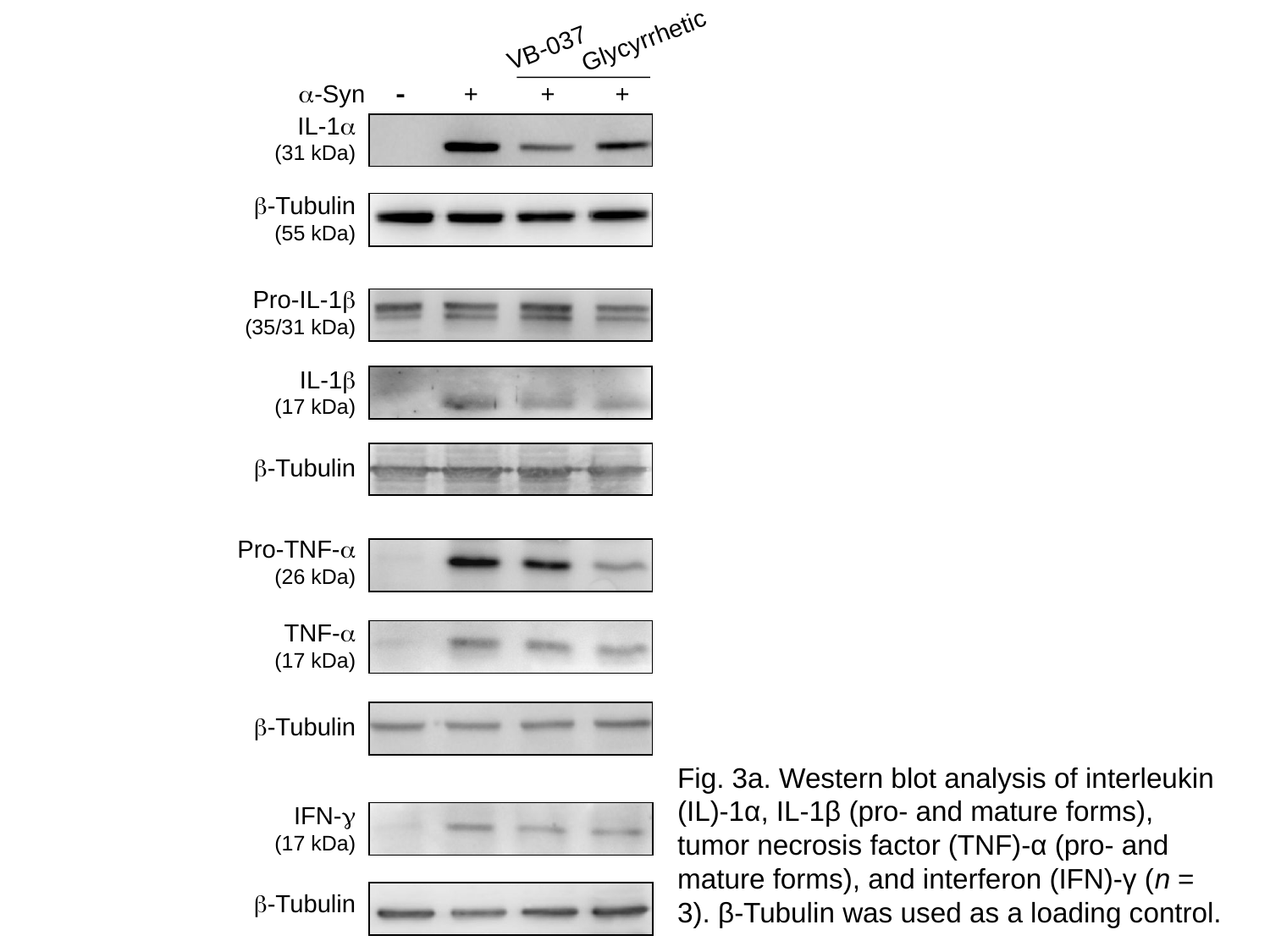

Glycyrrhetic
VB-037
a-Syn - + + +
IL-1a
(31 kDa)
b-Tubulin
(55 kDa)
Pro-IL-1b
(35/31 kDa)
IL-1b
(17 kDa)
b-Tubulin
Pro-TNF-a
(26 kDa)
TNF-a
(17 kDa)
b-Tubulin
IFN-g
(17 kDa)
b-Tubulin
Fig. 3a. Western blot analysis of interleukin (IL)-1α, IL-1β (pro- and mature forms), tumor necrosis factor (TNF)-α (pro- and mature forms), and interferon (IFN)-γ (n = 3). β-Tubulin was used as a loading control.

## Slide 4
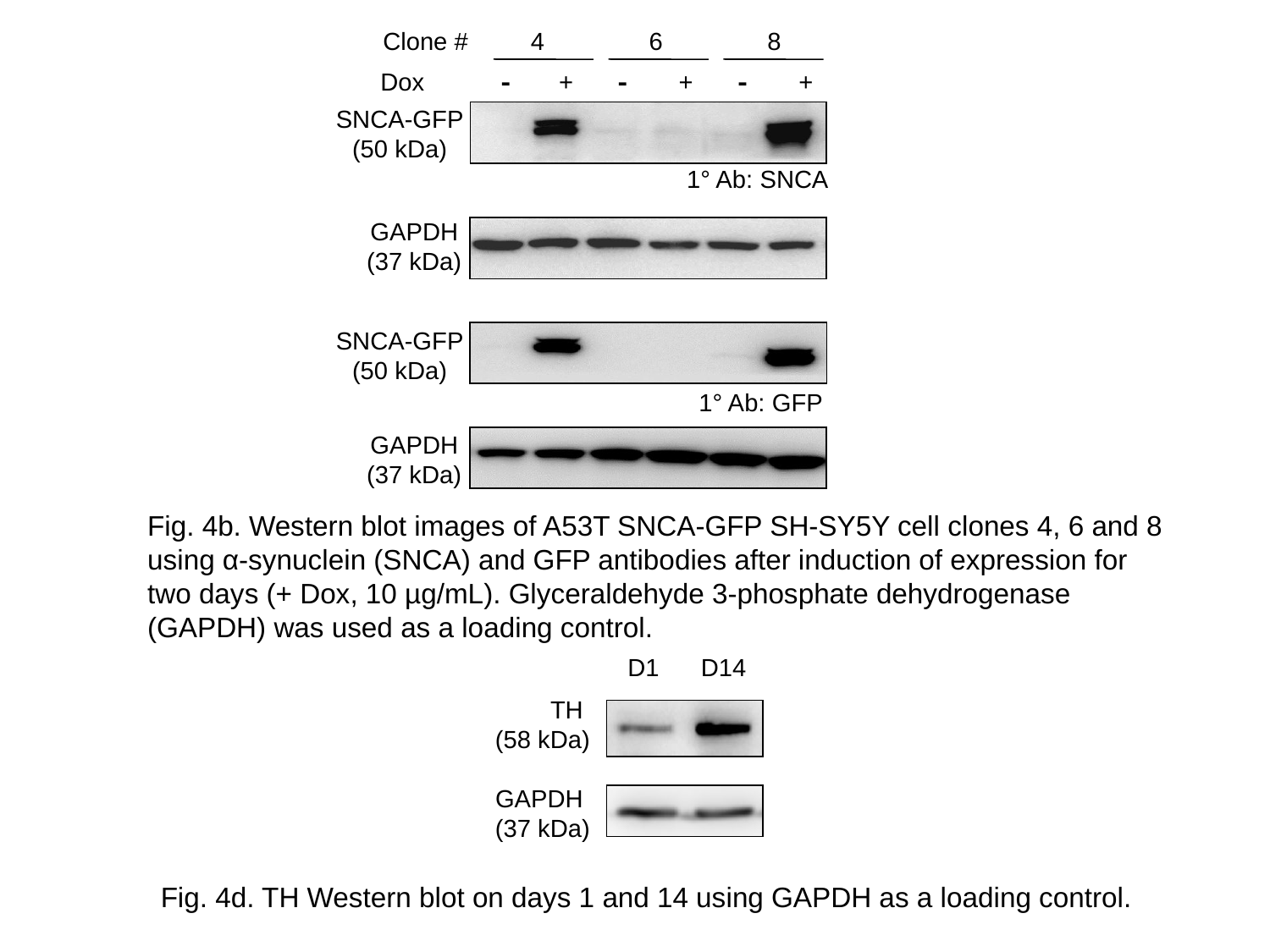

Clone # 4 6 8
Dox - + - + - +
SNCA-GFP
(50 kDa)
1° Ab: SNCA
GAPDH
(37 kDa)
SNCA-GFP
(50 kDa)
1° Ab: GFP
GAPDH
(37 kDa)
Fig. 4b. Western blot images of A53T SNCA-GFP SH-SY5Y cell clones 4, 6 and 8 using α-synuclein (SNCA) and GFP antibodies after induction of expression for two days (+ Dox, 10 µg/mL). Glyceraldehyde 3-phosphate dehydrogenase (GAPDH) was used as a loading control.
D1 D14
TH
(58 kDa)
GAPDH
(37 kDa)
Fig. 4d. TH Western blot on days 1 and 14 using GAPDH as a loading control.

## Slide 5
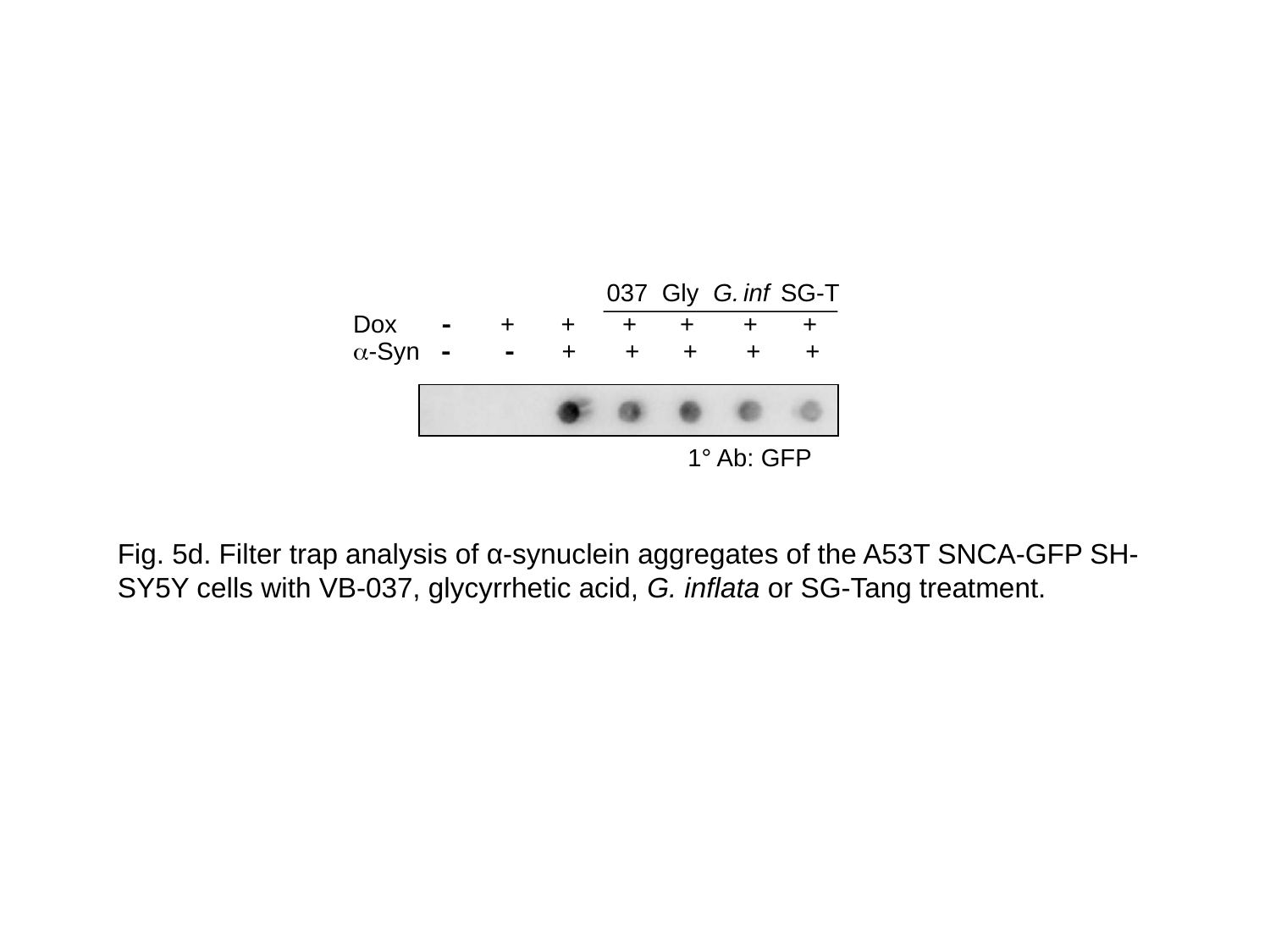

037 Gly G. inf SG-T
Dox - + + + + + +
a-Syn - - + + + + +
1° Ab: GFP
Fig. 5d. Filter trap analysis of α-synuclein aggregates of the A53T SNCA-GFP SH-SY5Y cells with VB-037, glycyrrhetic acid, G. inflata or SG-Tang treatment.

## Slide 6
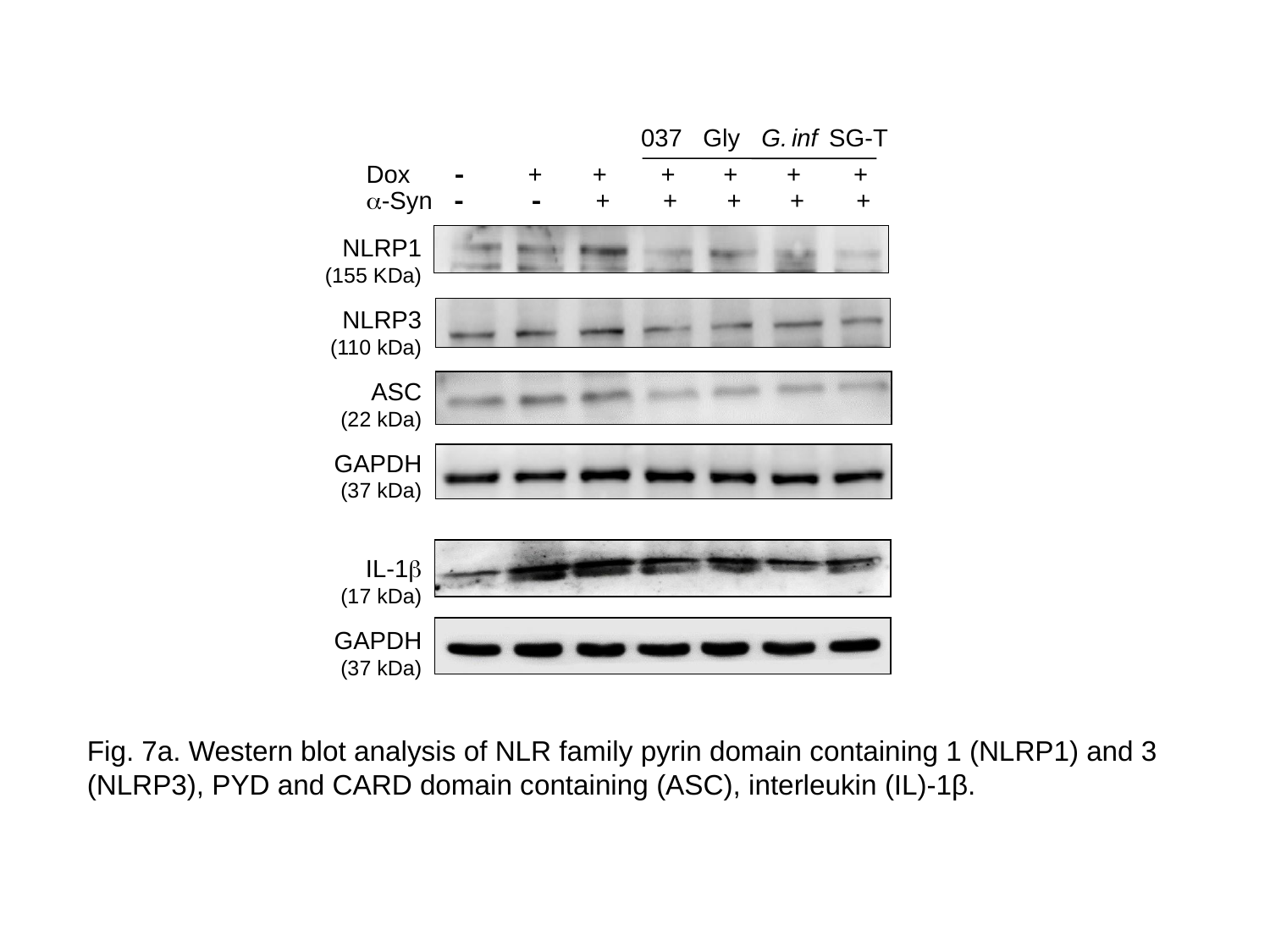

037 Gly G. inf SG-T
Dox - + + + + + +
a-Syn - - + + + + +
NLRP1
(155 KDa)
NLRP3
(110 kDa)
ASC
(22 kDa)
GAPDH
(37 kDa)
IL-1b
(17 kDa)
GAPDH
(37 kDa)
Fig. 7a. Western blot analysis of NLR family pyrin domain containing 1 (NLRP1) and 3 (NLRP3), PYD and CARD domain containing (ASC), interleukin (IL)-1β.

## Slide 7
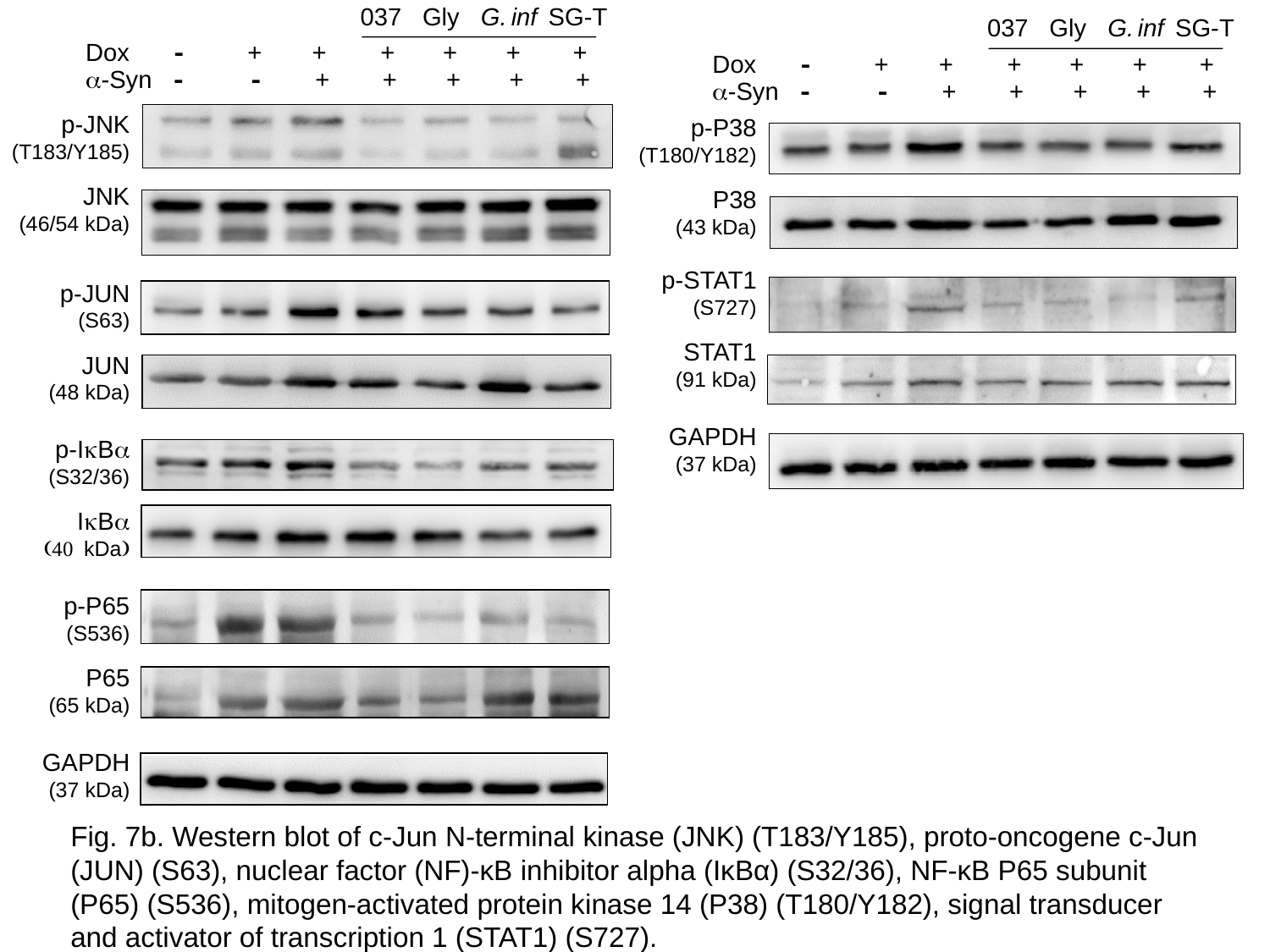

037 Gly G. inf SG-T
Dox - + + + + + +
a-Syn - - + + + + +
037 Gly G. inf SG-T
Dox - + + + + + +
a-Syn - - + + + + +
p-JNK
(T183/Y185)
JNK
(46/54 kDa)
p-JUN
(S63)
JUN
(48 kDa)
p-IkBa
(S32/36)
IkBa
(40 kDa)
p-P65
(S536)
P65
(65 kDa)
GAPDH
(37 kDa)
p-P38
(T180/Y182)
P38
(43 kDa)
p-STAT1
(S727)
STAT1
(91 kDa)
GAPDH
(37 kDa)
Fig. 7b. Western blot of c-Jun N-terminal kinase (JNK) (T183/Y185), proto-oncogene c-Jun (JUN) (S63), nuclear factor (NF)-κB inhibitor alpha (IκBα) (S32/36), NF-κB P65 subunit (P65) (S536), mitogen-activated protein kinase 14 (P38) (T180/Y182), signal transducer and activator of transcription 1 (STAT1) (S727).

## Slide 8
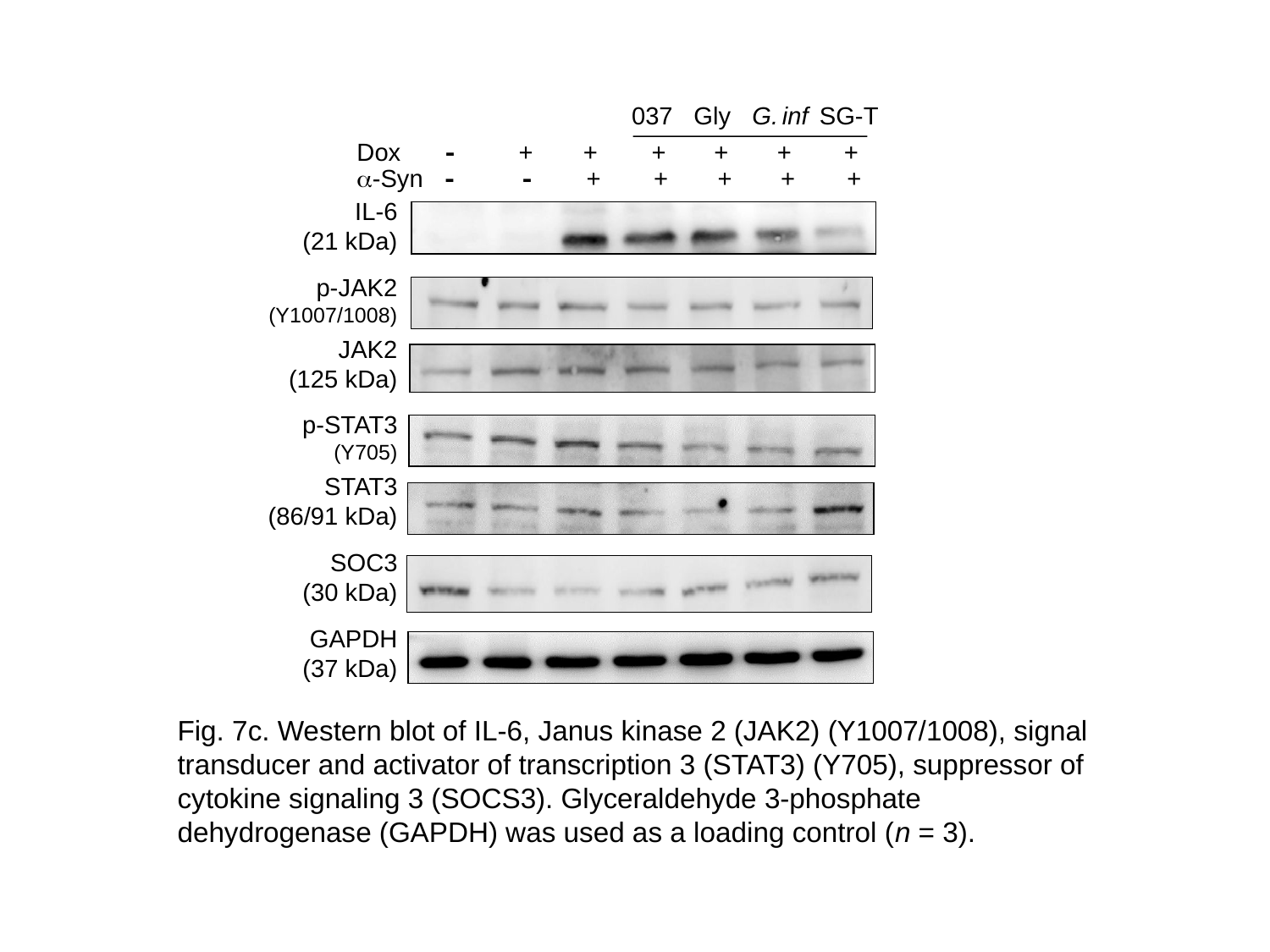

037 Gly G. inf SG-T
Dox - + + + + + +
a-Syn - - + + + + +
IL-6
(21 kDa)
p-JAK2
(Y1007/1008)
JAK2
(125 kDa)
p-STAT3
(Y705)
STAT3
(86/91 kDa)
SOC3
(30 kDa)
GAPDH
(37 kDa)
Fig. 7c. Western blot of IL-6, Janus kinase 2 (JAK2) (Y1007/1008), signal transducer and activator of transcription 3 (STAT3) (Y705), suppressor of cytokine signaling 3 (SOCS3). Glyceraldehyde 3-phosphate dehydrogenase (GAPDH) was used as a loading control (n = 3).
